# Resveratrol, a polyphenol phytoalexin, protects cardiomyocytes against anoxia/reoxygenation injury via the TLR4/NF-κB signaling pathway

**DOI:** 10.3892/ijmm.2012.885

**Published:** 2012-01-11

**Authors:** CUI ZHANG, GUOSHENG LIN, WEIGUO WAN, XUYON LI, BIN ZENG, BO YANG, CONGXIN HUANG

**Affiliations:** Department of Cardiology, Renmin Hospital of Wuhan University and Cardiovascular Research Institute of Wuhan University, Wuhan, P.R. China

**Keywords:** resveratrol, anoxia/reoxygenation, cardiomyocytes, Toll-like receptor 4, nuclear factor-κB

## Abstract

Previous studies indicate resveratrol pretreatment can protect cardiomyocytes. However, it is largely unknown whether resveratrol protects cardiomyocytes when applied at reperfusion. The purpose of this study was to investigate whether resveratrol given at reoxygenation could protect cardiomyocytes under the anoxia/reoxygenation (A/R) condition and to examine the underlying mechanism. In this study, primary cultures of neonatal rat cardiomyocytes were randomly distributed into three groups: control group, A/R group (cultured cardiomyocytes were subjected to 3 h anoxia followed by 2 h reoxygenation), and the resveratrol group (cardiomyocytes were subjected to 3 h anoxia/2 h reoxygenation, and 5, 10 or 20 μM resveratrol was applied 5 min after reoxygenation). In order to evaluate cardiomyocyte damage, cell viability, lactate dehydrogenase (LDH) release, caspase-3 activity, and apoptosis were analyzed by the cell counting kit (CCK)-8 assay, colorimetric method and flow cytometry, respectively. The mRNA and protein expression of Toll-like receptor 4 (TLR4) were detected by quantitative real-time PCR and western blot analysis. Nuclear factor-κB (NF-κB) p65 protein and I-κBα protein levels were also examined by western blot analysis. The levels of proinflammatory cytokines in the culture medium were assessed by enzyme-linked immunosorbent assay. We found that resveratrol prevented a reduction in cell viability, decreased the amount of LDH release, attenuated apoptotic cells and decreased caspase-3 activity induced by A/R in cardiomyocytes. Furthermore, resveratrol treatment significantly attenuated the TLR4 expression, inhibited NF-κB activation and reduced the levels of tumor necrosis factor (TNF)-α and interleukin (IL)-1β caused by A/R injury in the culture medium. Treatment with resveratrol shortly after the onset of reoxygenation improves cell survival and attenuates A/R-induced inflammatory response. This protection mechanism is possibly related to the TLR4/NF-κB signaling pathway.

## Introduction

Acute myocardial infarction (AMI) is the most common form of acute cardiac injury. The treatment of AMI has improved, however, AMI results in ischemic death of a large number of cardiomyocytes. Although early reperfusion of the ischemic myocardium in coronary artery infarction can rescue the agonal cardiac muscle, it causes subsequent myocardial ischemia-reperfusion (I/R) injury (1,2), which lowers the protective effect of reperfusion therapy. Myocardial I/R injury is a complex pathophysiological process that involves many kinds of factors and pathways. During the initiation and progression of I/R injury, apoptosis has been identified as providing an important molecular basis (3). Moreover, the inflammatory response is still considered to be a major cause of I/R-induced tissue injury (4).

Toll-like receptor 4 (TLR4) as a member of pattern recognition receptors is expressed and functional in cells of myeloid lineage. However, TLR4 is also detectable in non-professional immunocyte cell types, such as cardiomyocytes and microvascular endothelial cells (5). Recent studies have shown that TLR4 can induce apoptosis in several cell types (6–8). In addition, TLR4 plays an important role in the induction of the inflammatory response by recognition of several endogenous ligands associated with tissue injury (9). Activation of TLR4 induces the nuclear factor-κB (NF-κB)-dependent apoptosis and expression of proinflammatory cytokines (10,11). A previous report demonstrated that there is reduced myocardial injury and inflammation in TLR4-deficient mice after I/R (12).

Resveratrol (trans-3,4′,5-trihydroxystilbene), a naturally occurring polyphenol phytoalexin, is abundant in a wide variety of plant species, such as grapes, mulberries, peanuts and so on (13). Resveratrol has diverse biochemical and physiological actions, including antiplatelet (14), antiaging (15), antiapoptosis (16) and anti-inflammatory (17) actions. Recent studies have demonstrated that resveratrol can provide cardioprotective effects (18,19). It seems that resveratrol-mediated cardioprotection is achieved through the preconditioning effect rather than direct protection. Although preconditioning can effectively protect the heart from I/R injury, it can hardly be applied in the clinical setting of AMI because of the requirement for pretreatment. Therefore, it is significant to determine whether resveratrol applied at reperfusion can also protect the heart from I/R injury. If resveratrol is protective when given at reperfusion, it is interesting to define the potential cellular and molecular mechanisms underlying the protection.

Youn *et al* (20) reported that resveratrol could inhibit NF-κB activation induced by TLR4-mediated signaling in RAW264.7 cells. It has been demonstrated that the injury of cardiomyocytes induced by anoxia/reoxygenation (A/R) is a useful *in vitro* model to study myocardial I/R injury (21,22). Thus, in the present study, we first investigated whether resveratrol applied at reoxygenation could protect cardiomyocytes against A/R injury. Then we explored if the protective effect is exerted through the TLR4/NF-κB signaling pathway.

## Materials and methods

### Animals

Sprague-Dawley rats (1-3-days-old) were purchased from the Center of Experimental Animal in Wuhan University, China. All animals used in this study were cared for in accordance with the Guide for the Care and Use of Laboratory Animals published by the United States National Institute of Health (NIH publication no. 85-23, revised 1996), and all procedures were approved by the Committee of Experimental Animals of Wuhan University.

### Primary culture of neonatal rat cardiomyocytes

Primary cultures of neonatal rat cardiomyocytes were prepared from the ventricles of 1-3-day-old Sprague-Dawley rats, as described previously (23), with some modifications. Briefly, the hearts were harvested and placed in phosphate-buffered saline (calcium- and magnesium-free PBS: NaCl 137 mmol/l, Na_2_HPO_4_ 10.6 mmol/l, KH_2_PO_4_ 2.1 mmol/l, K_2_HPO_4_ 1.1 mmol/l, pH 7.4). The ventricles were minced into pieces approximately 1 mm^3^. The tissue fragments were dissociated by treatment with 0.125% trypsin 5 times at 37°C, then filtered and centrifuged for 10 min (120 × g), and finally resuspended in the culture medium, which consisted of Dulbecco’s modified Eagle’s medium (DMEM, Hyclone, Logan, UT) containing 10% fetal bovine serum (FBS, Invitrogen Corp., Carlsbad, CA), penicillin (100 U/ml) and streptomycin (100 μg/ml). Resuspended cells were then plated in a petri dish in a humidified incubator (5% CO_2_, 37°C) for 1.5 h to reduce fibroblast contamination. Non-adherent cells were counted with a hemocytometer and the final myocyte cultures were found to contain >90% cardiomyocytes. Subsequently the cells in the culture medium were transferred into 6-well gelatin-coated plates at a density of approximately 1×10^6^ cells/ml and incubated for 4 days before the experiment.

### A/R injury model

According to a previously described method (24), the *in vitro* model of A/R was used in this study. Briefly, the confluent beating cardiomyocytes in 6-well plates were exposed to anoxia for 3 h and then reoxygenated for 2 h. As a control, cardiomyocytes were initially perfused in normal Tyrode’s solution with a gas mixture of 95% O_2_-5% CO_2_ at 37°C, pH 7.4. To simulate anoxia, the Tyrode’s solution was switched to pH 6.8 at 37°C without glucose and then the cells were aerated with a gas mixture of 95% N_2_-5% CO_2_. To simulate reoxygenation, the cells were treated with normal Tyrode’s solution with a gas mixture of 95% O_2_-5% CO_2_. Anoxic conditions were obtained by equilibrating a small humidified plexiglass chamber containing cardiomyocytes with 95% N_2_ and 5% CO_2_ via a gas transfusive apparatus (Changjing Biotech Co., Beijing, China), which was confirmed by measuring chamber pO_2_ (chamber pO_2_ fell to 0 mmHg within 5 min after the initiation of perfusion with the anoxic gas). Reoxygenation was achieved by exposing cells to room air (CO_2_ incubator).

### Experimental groups and protocols

At the beginning of each experiment, the cells were rinsed in PBS, and the culture medium was replaced. Eighty percent confluent cardiomyocytes were randomly distributed into different experimental groups as follows, and each group included two parallel wells for three replicate experiments: i) control group: cardiomyocytes were incubated in aerobic Tyrode’s solution during the entire experimental period; ii) A/R group: cardiomyocytes were incubated in anaerobic Tyrode’s solution for 3 h anoxia followed by 2 h reoxygenation; 3) resveratrol group: cardiomyocytes were subjected to A/R as described above, and resveratrol (final concentrations: 5, 10 or 20 μM) was applied 5 min after reoxygenation and maintained throughout the experiment. Resveratrol (Sigma-Aldrich, St. Louis, MO) was freshly prepared as a 22.8 mg/ml solution in ethanol and then further diluted in cell culture medium. Cell viability and lactate dehydrogenase (LDH) activity were measured at the end of the reoxygenation times. Other measurements were performed after the cells were incubated at 37°C in a CO_2_ incubator for additional 24 h.

### Assay of cell viability

Cell viability was determined by the cell counting kit (CCK)-8 assay (Dojindo, Tokyo, Japan), and the experimental procedure was based on the manufacturer’s manual. The cardiomyocytes were seeded in 96-well plates at 1×10^4^ cells/well and incubated with different concentrations of resveratrol alone (0, 5, 10, 20 μM) for 48 h, or after 3 h anoxia. These cells were treated with different concentrations of resveratrol (0, 5, 10, 20 μM) that was applied 5 min after 2 h reoxygenation. After experimental treatment, 10 μl of WST-8 solution (2-(2-methoxy-4-nitrophenyl)-3-(4-nitrophenyl)-5-(2,4-disulfophenyl)-2H-tetrazolium, monosodium salt) was added to each well, and the cardiomyocytes were incubated for an additional 2 h at 37°C. The absorbance of each well at 450 nm was measured with a reference at 630 nm using a microplate reader (Bio-Rad Laboratories, Hercules, CA). The percentage of cell viability was calculated by the following formula: % cell viability = (mean absorbance in test wells)/(mean absorbance in control well) × 100.

### Assay of lactate dehydrogenase (LDH) activity

The extent of cellular injury was monitored by measuring LDH release. According to the manufacturer’s instruction, 100 μl of culture medium was taken to assess LDH activity using a commercial kit (JianCheng Bioengineering Institute, Nanjing, China) with a spectrophotometer.

### Flow cytometric analysis of apoptosis

Apoptosis was assessed by flow cytometric analysis of Annexin V and propidium lodide (PI) double staining. The cardiomyocytes were seeded in 6-well plates at approximately 2×10^4^ cells/well. After treatment, cells were centrifuged to remove the medium, rinsed in PBS, and suspended in 100 μl of 1X binding buffer (10 mM HEPES, 140 mM NaCl, and 2.5 mM CaCl_2_) to be stained with Annexin V and PI according to the manufacturer’s instructions (BioVision, Inc., Palo Alto, CA). Stained cells were analyzed using a FACStar plus flow cytometer (Becton-Dickinson, San Jose, CA) in the FL1-H and FL2-H channels.

### Measurement of caspase-3 activity

Caspase-3 activity was evaluated by using a commercialized caspase-3 assay kit (Biovision, Inc.). Approximately 1×10^6^ cells were harvested by centrifugation, and the pellet was resuspended in lysis buffer. Protein levels were determined with the bicinchoninic acid assay (Beyotime Biotechnology, Shanghai, China). As described in the manufacturer’s instructions, aliquots of protein (10 μl) were incubated with 10 μl of synthetic peptide substrate Ac-DEVD-pNA in a total volume of 100 μl at 37°C for 2 h to detect caspase-3 activity. Caspase-3 activity was expressed as optical density. The absorbance at 405 nm of the released pNA was monitored in a spectrophotometer.

### Quantitative real-time PCR analysis

Total-RNA was prepared from cells with TRIzol reagent (Invitrogen Corp.) and reversely transcribed to produce cDNA from total-RNA with oligo(dt). The expression of candidate genes were measured by quantitative real-time PCR analysis using a SYBR-Green-based assays kit (Invitrogen Corp.) to amplify the fragments according to the manufacturer’s instructions. The RT-PCR conditions were 42°C/15 min, 95°C/2 min for reverse transcription; 95°C/30 sec, 58.9°C (TLR4) or 60°C (GAPDH)/30 sec, and 72°C/60 sec, over 40 cycles for polymerase chain reaction. Level of TLR4 mRNA was calculated based on the method of 2^−ΔΔCT^ between the intervening group and the control group. GAPDH was used as an internal control, and the comparative threshold method was used to assess the relative abundance of TLR4 mRNA. The specific primer sequence of the selected genes were: TLR4, sense, 5′-AGCCATTGCTGCCAACATCA-3′ and antisense primer, 5′-GCCAGAGCTACTCAGAAAC-3′; GAPDH, sense, 5′-GACAACTTTGGCTCGTGGA-3′ and antisense primer, 5′-ATGCAGGGGTTCTGG-3′. Primers were synthesized by Shanghai Sangon Biological Engineering Technology Company Limited (China). The correctness of the gene order was proven in GenBank.

### Western blot analysis

Membranous, cytoplasmic and nuclear extracts were prepared for western blot analysis of TLR4 (membranous), I-κBα (cytoplasmic) and NF-κBp65 (nuclear) expression, using Membranous, Cytoplasmic and Nuclear Extraction Reagents (Pierce Biotechnology, Inc., Rockford, IL). Protein concentration was determined by the bicinchoninic acid protein assay (Beyotime Biotechnology). Equal amounts (50 μg) of denatured proteins were separated on 10% SDS-polyacrylamide gels and transferred to nitrocellulose membrane. The membranes were blocked with 5% non-fat dry milk in TBST (containing 0.05% Tween-20), and incubated overnight at 4°C with the primary antibody (TLR4, 1:1,000, Cell Signaling Technology, Inc., Beverly, MA; I-κBα, 1:500, Santa Cruz Biotechnology, Inc., Santa Cruz, CA; NF-κBp65, 1:500, Santa Cruz Biotechnology, Inc.). Then the blots were washed and incubated with horseradish peroxidase-conjugated secondary antibody (goat anti-rabbit IgG, 1:2,000, Beyotime Biotechnology) for 1 h at room temperature. Immunoreactivity was enhanced with a chemiluminescence kit (Beyotime Biotechnology) and exposed to film. β-actin was used as an internal control to correct the variations of different samples. The density of bands on western blots was quantified by using a Bio-Rad image system (Hercules, CA).

### Enzyme-linked immunosorbent assay

The levels of tumor necrosis factor (TNF)-α and interleukin (IL)-1β in the culture medium were measured by enzyme-linked immunosorbent assay (ELISA), using commercially available kits (Zhong Shan-Golden Bridge Biological Technology Co., Beijing, China) according to the manufacturer’s instructions.

### Statistical analysis

Data are expressed as means ± SD. Statistical analyses of data were performed by one-way ANOVA followed by the Student-Newman-Keuls test. A value of P<0.05 was considered to be statistically significant. All data analyses were conducted with the SPSS 13.0 software package (SPSS, Inc., Chicago, IL).

## Results

### Effect of resveratrol on A/R-induced cell damage in cardiomyocytes

**Figure 1:**
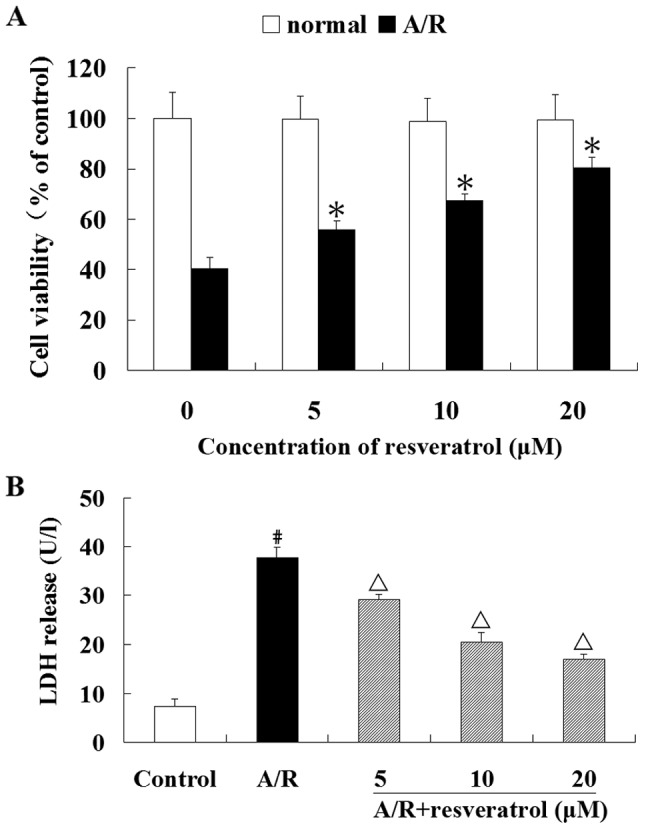
Resveratrol protects against A/R-induced cell death in cardiomyocytes. (A) After the cardiomyocytes were incubated with resveratrol (0, 5, 10, 20 μM) under normal condition or undergoing A/R, the viability was measured by the CCK-8 assay. (B) Effects of resveratrol (5, 10, 20 μM) on LDH activity in cardiomyocytes subjected to A/R. Date are expressed as means ± SD (n=6). ^*^P<0.05 vs. untreated control group; ^#^P<0.05 vs. control group; ^Δ^P< 0.05 vs. A/R group.

To observe the cytotoxicity of resveratrol on cardiomyocytes, the cells were exposed to different concentrations of resveratrol (0, 5, 10 or 20 μM) for 48 h, and the CCK-8 assay showed no loss of cell viability (Fig. 1A). Therefore, we used resveratrol at the concentrations of 5, 10 or 20 μM for our subsequent studies. Resveratrol (5, 10 or 20 μM) significantly prevented the loss of cardiomyocyte viability that resulted from A/R induction (P<0.05) (Fig. 1A). Furthermore, resveratrol (5, 10 or 20 μM) significantly suppressed the release of LDH in cardiomyocytes that had undergone A/R (P<0.05) (Fig. 1B).

**Figure 2:**
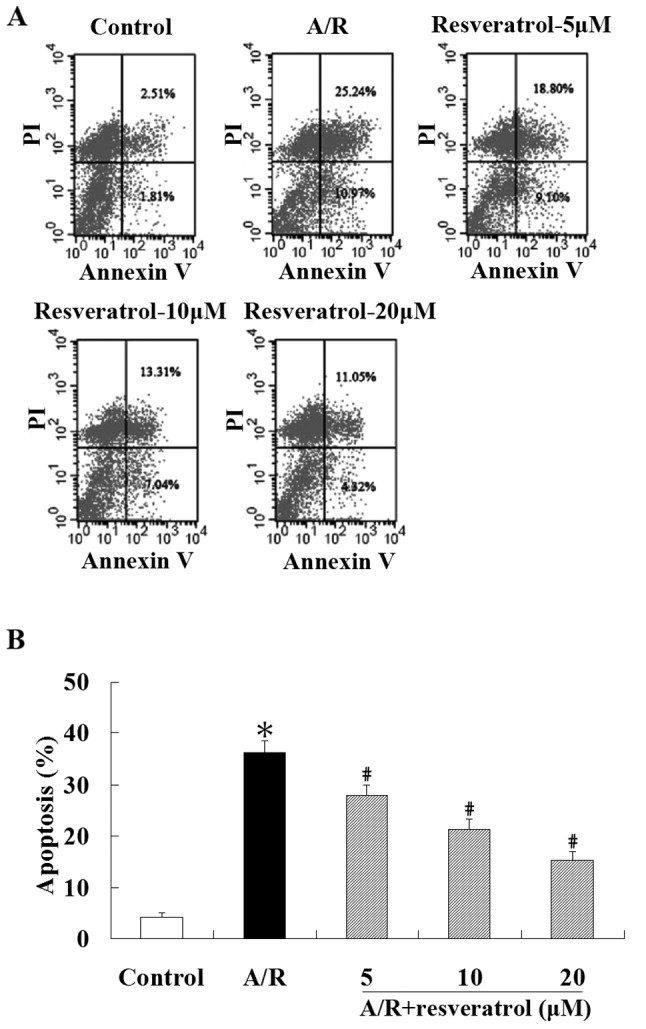
Effects of resveratrol on the apoptosis in cardiomyocytes subjected to A/R. (A) Detection of apoptotic cells by Annexin V and PI double staining. Cardiomyocytes were treated with resveratrol (0, 5, 10, 20 μM), stained with Annexin V and PI labeling and analyzed by flow cytometry. (B) Column bar graph of apoptosis. Date are expressed as means ± SD (n=6). ^*^P<0.05 vs. the control group; ^#^P<0.05 vs. the A/R group.

Flow cytometric analysis was used to quantify the rate of cell apoptosis. Spontaneous apoptosis was low in cardiomyocytes under control condition, yet stimulation induced by A/R in cardiomyocytes led to enhanced apoptosis (P<0.05). Treatment with resveratrol (5, 10 or 20 μM) showed a significant resistance in apoptosis in cardiomyocytes undergoing A/R (P<0.05) (Fig. 2). These results suggest that resveratrol is a potent cardioprotective agent against A/R injury.

### Effect of resveratrol on the activity of caspase-3 induced by A/R in cardiomyocytes

**Figure 3:**
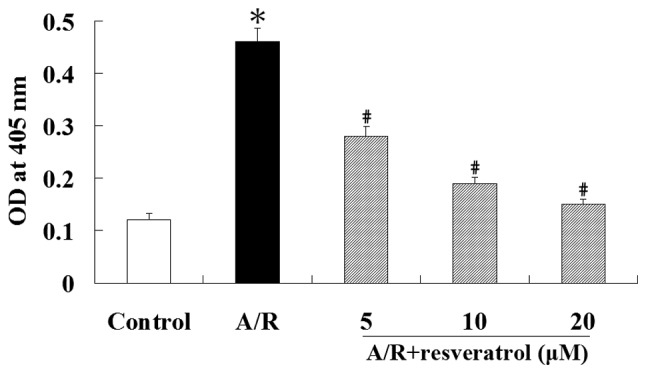
Effect of resveratrol on caspase-3 activity in cardiomyocytes exposed to A/R. Caspase-3 activity was measured as described in Materials and methods. Data are expressed as means ± SD (n=6). ^*^P<0.05 vs. the control group; ^#^P<0.05 vs. the A/R group.

Caspase-3 activation is unique to apoptosis as it does not occur in other forms of cell death and provides strong evidence for the presence of apoptosis. Therefore, we examined the activity of caspase-3 using the synthetic peptide substrate Ac-DEVD-pNA. Caspase-3 activation was increased during A/R (P<0.05). However, resveratrol significantly inhibited the activation of caspase-3 induced by A/R (P<0.05) (Fig. 3). These results suggest that resveratrol protects against A/R-induced apoptosis associated with the inhibition of caspase-3 in cardiomyocytes.

### Effect of resveratrol on TLR4 expression in cardiomyocytes undergoing A/R

**Figure 4:**
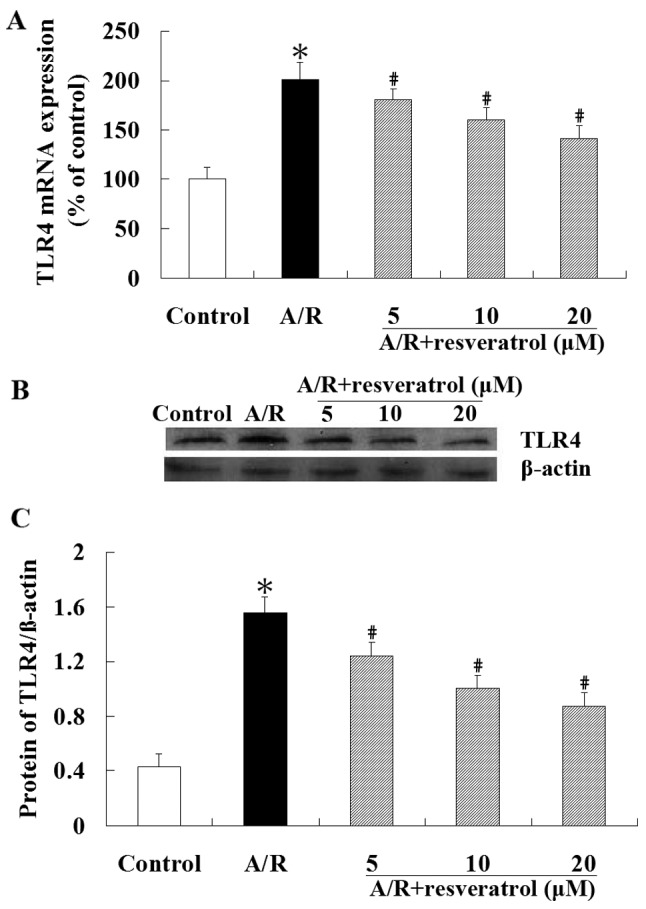
TLR4 expression in the different experimental groups. Resveratrol inhibited the expression of TLR4 mRNA and protein in cardiomyocytes undergoing A/R. (A) TLR4 mRNA expression was examined by real-time PCR. The results were expressed as relative expression to GAPDH and plotted as ratio of the control group. (B) Representative western blot analyses of TLR4 and β-actin expression. (C) Bands were analyzed and quantified by densitometry and the TLR4/β-actin ratio was evaluated. Date are expressed as means ± SD (n=6). ^*^P<0.05 vs. the control group; ^#^P<0.05 vs. the A/R group.

Real-time RT-PCR revealed that the expression of TLR4 mRNA was significantly increased in cardiomyocytes undergoing A/R (P<0.05). However, compared with the A/R group, treatment with resveratrol (5, 10 or 20 μM) produced a significant reduction of TLR4 mRNA expression (P<0.05) (Fig. 4A). Similar to the qRT-PCR result, western blot analysis showed that the TLR4 protein level was significantly lower in resveratrol group compared with the A/R group (P<0.05) (Fig. 4B).

### Effect of resveratrol on NF-κB translocation in cardiomyocytes undergoing A/R

**Figure 5:**
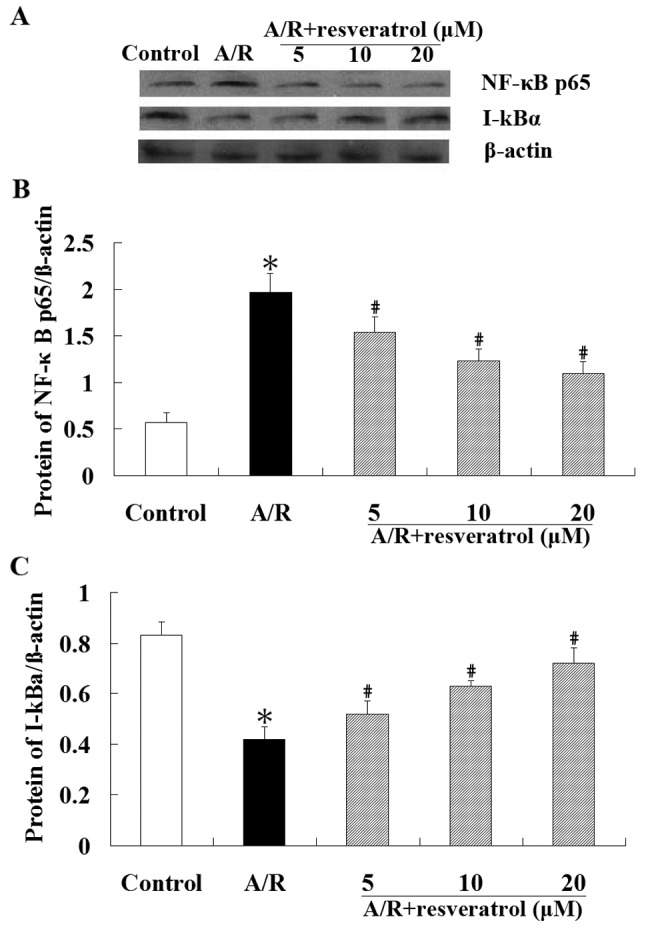
Effects of resveratrol on NF-κB nuclear translocation in cardiomyocytes exposed to A/R. (A) Representative western blot analyses of NF-κB p65, I-κBα and β-actin. (B) Bands were analyzed and quantified by densitometry and the NF-κBp65/β-actin ratio was evaluated. (C) Bands were analyzed and quantified by densitometry and the I-κBα/β-actin ratio was evaluated. Date are expressed as means ± SD (n=6). ^*^P<0.05 vs. the control group; ^#^P<0.05 vs. the A/R group.

The western blot analysis was performed to explore whether resveratrol has an effect on NF-κB translocation. These results showed that A/R induced nuclear translocation of NF-κBp65 protein in cardiomyocytes, which was markedly attenuated by resveratrol (P<0.05). Cytoplasmic I-κBα protein levels were significantly higher (P<0.05) in resveratrol groups than those in A/R group. As demonstrated in Fig. 5, A/R induced an obvious NF-κB nuclear translocation in an IκBα-dependent manner. Resveratrol (5, 10 or 20 μM) treatment inhibited the degradation of I-κBα, and blocked the translocation of NF-κB into the nucleus.

### Resveratrol inhibits the production of TNF-α and IL-1β induced by A/R in cardiomyocytes

**Figure 6:**
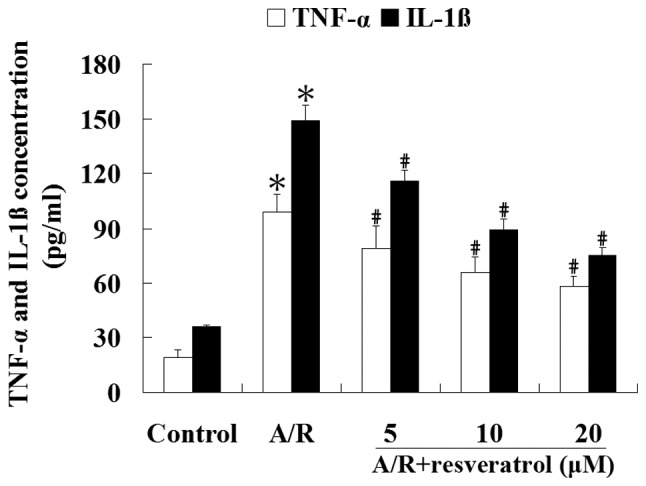
Effects of resveratrol on TNF-α and IL-1 β production in cardiomyocytes. ELISA analyzed the concentrations of TNF-α and IL-1 β in the culture medium. Date are expressed as means ± SD (n=6). ^*^P<0.05 vs. the control group; ^#^P<0.05 vs. the A/R group.

ELISA was used to analyze the concentrations of TNF-α and IL-1β in the culture medium. Cardiomyocytes subjected to A/R had an increase of TNF-α and IL-1β contentrations in the culture medium compared with the control group (P<0.05). Treatment with resveratrol (5, 10 or 20 μM) produced a significant reduction of TNF-α and IL-1β concentrations in the culture medium undergoing A/R (P<0.05) (Fig. 6). These results suggest that resveratrol can significantly attenuate the levels of proinflammatory cytokines induced by A/R in cardiomyocytes (P<0.05).

## Discussion

The principal findings of the present study were that i) the toxicity of resveratrol (5, 10 or 20 μM) on cardiomyocytes was negligible and resveratrol applied shortly after the onset of reoxygenation markedly suppressed the decrease of cell viability resulting from A/R, and reduced the levels of LDH in the culture medium of cardiomyocytes; ii) Resveratrol administration decreased apoptotic cardiomyocytes, caspase-3 activity and attenuated the level of proinflammatory cytokines (TNF-α and IL-1β) induced by A/R in cardiomyocytes; iii) treatment with resveratrol downregulated the TLR4 expression and blocked NF-κB translocation from the cytoplasm to the nucleus in an IκBα-dependent manner. Our data showed that resveratrol exerted the protective effects by inhibiting cell death and inflammation in cardiomyocytes undergoing A/R, which might be associated with the TLR4/NF-κB signaling pathway.

The measures of cell viability and level of LDH are usually used as indicators of cardiomyocyte injury. In the present study, we found that the cell viability was markedly decreased and the level of LDH in the culture medium was increased in cardiomyocytes undergoing A/R, which indicated severe cardiomyocyte membrane damage. However, resveratrol (5, 10 or 20 μM) significantly prevented A/R-induced cell damage in cardiomyocytes confirmed by improved cell viability and reduced LDH activity, implying that resveratrol can protect cardiomyocytes.

Shortly after the onset of ischemia, the apoptotic process of cardiomyocytes is initiated and subsequently cells undergo necrosis. Paradoxically, reperfusion itself may lead to necrosis and accelerate the process of apoptosis in cardiomyocytes (2). Extracellular stimuli can trigger apoptosis via the death receptor and mitochondrial mediated pathways (25). The activation of caspase cascade is an important mechanism in regulating cell apoptosis (26). As one of the key effectors, the activity of caspase-3 was found to increase after I/R injury (27). Consistent with a previous report, in this study, we found that stimulation induced by A/R in cardiomyocytes resulted in enhanced apoptosis and increased caspase-3 activation. Treatment with resveratrol shortly after the onset of reoxygenation significantly reduced the number of apoptotic cells and attenuated caspase-3 activity, providing a evidence that resveratrol can protect cardiomyocytes against A/R injury by antiapoptosis.

In addition, the inflammatory response plays a crucial role in myocardial I/R injury. In our study, we found an increase of proinflammatory cytokines (TNF-α and IL-1β) concentrations in the culture medium of cardiomyocytes subjected to A/R. However, resveratrol applied shortly after the onset of reoxygenation markedly suppressed the levels of TNF-α and IL-1β in the culture medium. The result showed that resveratrol can also protect cardiomyocytes against A/R injury via an anti-inflammatory response.

In this study, another important finding was that resveratrol applied shortly after the onset of reoxygenation decreased TLR4 mRNA and protein expression, which were upregulated in cardiomyocytes undergoing A/R. Increasing evidence has indicated that TLR4 has a central role in myocardial I/R injury through TLR4 signaling (28,29). Endogenous ligands released from damaged cells or tissue fragments seem to initiate cellular apoptosis and the inflammatory response by inducing TLR4 signaling (8,30,31). TLR4 signaling could be downregulated through c-Jun-N-terminal kinases (JNKs), p38 kinases, and the NF-κB pathway (32). As a preformed trimeric complex (mainly consisting of the proteins p50 and p65), NF-κB interacts with the inhibitory proteins, I-κBα in the cytoplasm. IKKβ (a kinase) phosphorylates I-κBα resulting in the subsequent degradation of I-κBα. When liberated from the inhibition, NF-κB translocates to the nucleus, where it remains activated (33). NF-κB is an important transcription factor in TLR4-mediated signaling pathway regulating genes encoding proteins implicated in apoptosis and inflammation (34,35). In our study, we found that A/R induced NF-κB nuclear translocation in an IκBα-dependent manner, enhanced cardiomyocyte apoptosis, increased caspase-3 activation and the production of proinflammatory cytokines (TNF-α, IL-1β) accompanied with the upregulation of TLR4 expression, implying that TLR4 may play an important role in triggering apoptosis and the inflammatory response in the process of myocardial A/R.

Increasing evidence exists supporting that at a lower dose resveratrol acts as an antiapoptotic agent, providing cardioprotection by increasing the expression of cell survival proteins, improving post-ischemic ventricular recovery and reducing myocardial infarct size and cardiomyocyte apoptosis compared to the control. However, at higher dose, resveratrol acts as a pro-apoptotic compound, inducing apoptosis by exerting a death signal, increases myocardial infarct size and the number of apoptotic cells (36,37). Consistent with these previous studies, our data demonstrate that resveratrol protected cardiomyocytes at a relatively low dose (5–20 μM). Although direct beneficial effects of resveratrol on cardiomyocytes at a lower dose have already been reported, the underlying mechanisms remain poorly understood. Das and Maulik (38) reported that the antiapoptotic and antiinflammatory effects of resveratrol in cardioprotection were related to a number of signaling pathways. Interestingly, our study found that resveratrol applied shortly after the onset of reoxygenation could improve cell survival and attenuate A/R-induced inflammatory response through the downregulation of TLR4/NF-κB signaling pathway. Therefore, we presumed that resveratrol protects cardiomyocytes against A/R injury by decreasing the number of apoptotic cardiomyocytes and attenuating the inflammatory response under A/R conditions as an *in vitro* I/R model, which might be mediated in part by TLR4/NF-κB signaling pathway.

In conclusion, our data provide new insight to understand the role of resveratrol in protecting cardiomyocytes against A/R injury. The increased number of apoptotic cardiomyocytes and the production of TNF-α and IL-1β were associated with the elevated expression of TLR4 and enhanced NF-κB activation in the cardiomyocyte A/R model, which could be inhibited by resveratrol administered shortly after the onset of reoxygenation in cardiomyocytes. Thus, our results suggest that as an adjuvant therapeutic approach, resveratrol applied at reperfusion may constitute a new strategy for myocardial I/R injury at least in part via the TLR4-mediated NF-κB signaling pathway. In the future, *in vivo* studies will be carried out to determine the potentially protective role of resveratrol for the treatment of myocardial I/R injury.
